# Supplementary material for: HIVprotI: an integrated web based platform for prediction and design of HIV proteins inhibitors
Source: J Cheminform. 2018 Mar 9;10:12. doi: 10.1186/s13321-018-0266-y (PMC5845081; doi:10.1186/s13321-018-0266-y)
Supplement: Supplementary file 2 — Additional file 2. Source code of HIVProtI web server. [file 13321_2018_266_MOESM2_ESM.zip › HIVprotI_Source-code/hivprotI/jsmol/testEPFL.htm]

C8H10N4O2
APtclcactv12041317423D 0 0.00000 0.00000
24 25 0 0 0 0 0 0 0 0999 V2000
1.3120 -1.0479 0.0025 N 0 0 0 0 0 0 0 0 0 0 0 0
2.2465 -2.1762 0.0031 C 0 0 0 0 0 0 0 0 0 0 0 0
1.7906 0.2081 0.0010 C 0 0 0 0 0 0 0 0 0 0 0 0
2.9938 0.3838 0.0002 O 0 0 0 0 0 0 0 0 0 0 0 0
0.9714 1.2767 -0.0001 N 0 0 0 0 0 0 0 0 0 0 0 0
1.5339 2.6294 -0.0017 C 0 0 0 0 0 0 0 0 0 0 0 0
-0.4026 1.0989 -0.0001 C 0 0 0 0 0 0 0 0 0 0 0 0
-1.4446 1.9342 -0.0010 N 0 0 0 0 0 0 0 0 0 0 0 0
-2.5608 1.2510 -0.0000 C 0 0 0 0 0 0 0 0 0 0 0 0
-2.2862 -0.0680 0.0015 N 0 0 0 0 0 0 0 0 0 0 0 0
-3.2614 -1.1612 0.0029 C 0 0 0 0 0 0 0 0 0 0 0 0
-0.9114 -0.1939 0.0014 C 0 0 0 0 0 0 0 0 0 0 0 0
-0.0163 -1.2853 -0.0022 C 0 0 0 0 0 0 0 0 0 0 0 0
-0.4380 -2.4279 -0.0068 O 0 0 0 0 0 0 0 0 0 0 0 0
3.2697 -1.8004 0.0022 H 0 0 0 0 0 0 0 0 0 0 0 0
2.0830 -2.7828 0.8938 H 0 0 0 0 0 0 0 0 0 0 0 0
2.0821 -2.7846 -0.8862 H 0 0 0 0 0 0 0 0 0 0 0 0
2.6223 2.5703 -0.0019 H 0 0 0 0 0 0 0 0 0 0 0 0
1.1987 3.1611 -0.8923 H 0 0 0 0 0 0 0 0 0 0 0 0
1.1990 3.1632 0.8877 H 0 0 0 0 0 0 0 0 0 0 0 0
-3.5520 1.6797 -0.0001 H 0 0 0 0 0 0 0 0 0 0 0 0
-3.5037 -1.4333 -1.0244 H 0 0 0 0 0 0 0 0 0 0 0 0
-2.8389 -2.0244 0.5173 H 0 0 0 0 0 0 0 0 0 0 0 0
-4.1672 -0.8395 0.5168 H 0 0 0 0 0 0 0 0 0 0 0 0
1 2 1 0 0 0 0
1 3 1 0 0 0 0
3 4 2 0 0 0 0
3 5 1 0 0 0 0
5 6 1 0 0 0 0
5 7 1 0 0 0 0
7 8 1 0 0 0 0
8 9 2 0 0 0 0
9 10 1 0 0 0 0
10 11 1 0 0 0 0
10 12 1 0 0 0 0
7 12 2 0 0 0 0
12 13 1 0 0 0 0
1 13 1 0 0 0 0
13 14 2 0 0 0 0
2 15 1 0 0 0 0
2 16 1 0 0 0 0
2 17 1 0 0 0 0
6 18 1 0 0 0 0
6 19 1 0 0 0 0
6 20 1 0 0 0 0
9 21 1 0 0 0 0
11 22 1 0 0 0 0
11 23 1 0 0 0 0
11 24 1 0 0 0 0
M END
$$$$
